# Operationalizing community engagement evaluation: A structured and scalable approach using the RE-AIM framework and net effects diagrams

**DOI:** 10.1017/cts.2025.10190

**Published:** 2025-10-29

**Authors:** Brian Do-Golden, Nicole Wolfe, Nicole M.G. Maccalla, James Settles, Michele D. Kipke

**Affiliations:** 1 University of Southern California, Southern California Clinical and Translational Science Institute, Los Angeles, CA, US; 2 University of Southern California, Office of the Senior Vice President of Health Affairs, Los Angeles, CA, US; 3 Children’s Hospital of Los Angeles, Department of Pediatrics Los Angeles, CA, US

**Keywords:** Community engagement (CE), implementation science (IS), re-aim framework, evidence-based program adaptation, community development evaluation

## Abstract

**Introduction:**

Community engagement (CE) is essential in Clinical and Translational Science (CTS), yet its evaluation remains inconsistent and often lacks standardization. The RE-AIM framework (Reach, Effectiveness, Adoption, Implementation, Maintenance) offers a promising structure for evaluating CE efforts, but its application in dynamic, community-based contexts is often limited by data variability and implementation complexity.

**Methods:**

We developed and applied a seven-step, structured, and replicable approach to operationalizing RE-AIM for program evaluation. This method includes the use of tailored RE-AIM subdomains, standardized scoring systems, and visual analytics through Net Effects Diagrams.

**Results:**

We applied this framework to our community-based health education workshops delivered in English and Spanish across Los Angeles, using participant surveys and facilitator feedback data. The operationalized framework enabled consistent assessment and comparison between language groups. Spanish-language workshops outperformed English-language workshops (ELWs) in measures of attendance, participant satisfaction, and short-term effectiveness. Visualizations using Net Effects Diagrams facilitated collaboration among stakeholders to interpret program outputs and outcomes, supporting actionable insights for program adaptation. Differences between workshop groups will inform changes to recruitment and content delivery strategies in ELWs.

**Conclusions:**

This approach offers a transparent, scalable, and context-sensitive method for assessing CE programs. It supports data-driven decision-making, continuous program improvement, and stakeholder engagement. While developed for CE initiatives, the method is broadly adaptable to other community and public health programs. Future efforts will include expanded outcome tracking, integration into dashboards, and dissemination as a toolkit for broader adoption within and beyond the CTS Award network.

## Introduction

The Clinical and Translational Science Award (CTSA) Program in the United States, funded by the National Institutes of Health, supports the advancement of efficient research translation, speeding up the process of taking the discoveries made in laboratories, clinics, and communities to inform and improve processes and policies [1]. Community engagement (CE) programs are an essential and required element of each of the 60+ CTSA hubs, recognizing that meaningful engagement with communities enhances research relevance, accessibility, and impact [2]. CE is central to CTS, particularly for building and maintaining trust in populations that have historically lower rates of participating in research and accessing the healthcare system [1].

The CE Core within the Southern California Clinical and Translational Science Institute (SC CTSI) serves as a critical bridge between the community, researchers, and academic institutions. We focus on fostering meaningful engagement with the communities in South, Central, and the Eastside of Los Angeles, communities that have long faced limited access to healthcare, disproportionately higher rates of chronic disease, and lower rates of participation in clinical research.

The SC CTSI CE Core implements a range of initiatives designed to promote health education, research literacy, and bidirectional collaboration between community members and academic researchers. One key component is our education and training program, which delivers in-person and virtual workshops on health and research-related topics that aim to impact community well-being, including nutrition, mental health, diabetes, and other chronic diseases. Additionally, we offer a well-rounded research training program tailored for community health workers/promotores de salud, equipping them with the knowledge and skills to support health research and outreach efforts in their communities. Beyond community education, we also provide consultations to researchers, guiding them in integrating CE frameworks and community-based participatory research methodologies into their work.

CE efforts are vital for addressing differences in health outcomes and developing and maintaining key partnerships that bridge the gap between scientific discovery and the well-being of communities [3]. Despite its importance, evaluating CE efforts remains challenging due to the lack of robust, standardized, replicable frameworks. This makes it difficult to identify best practices, compare outcomes, and maintain effective engagement approaches [4]. Traditionally, the evaluation of CE work often relies on academic outcomes such as publications rather than assessing the real-world impact of CE activities on communities, partnerships, and translational research outcomes [5]. Therefore, a robust, standardized evaluation framework is needed that encapsulates the *Reach* and *Effectiveness* of CE initiatives as well as their *Adoption*, *Implementation*, and *Maintenance*.

Given the complexity and variability of CE activities, an evaluation framework must assess impact at multiple levels and offer insights that facilitate practical programmatic decision-making. Implementation science (IS) has been used for CE evaluation to provide a coordinated structure for assessing real-world implementation and identifying components that lead to success [6]. Traditionally focusing on shrinking the gap between research discoveries and their practical application in community and clinical settings, IS has also been used to improve adoption and effectiveness of evidence-based practices in behavior interventions, healthcare delivery, and public health [7,8]. Within the CTS context, IS principles have accelerated the dissemination and implementation of research findings, translating clinical advancements into meaningful community and population-level health improvements [9]. Despite its integration into CTSA hubs’ planning and implementation, IS frameworks remain underutilized for evaluating CTSA hubs’ CE initiatives, limiting the ability to fully assess their impact [10].

Among IS frameworks, RE-AIM allows for the adaptability of community-based research and its ability to capture implementation, engagement, and effectiveness outcomes in a standardized manner. This has proven to be valuable in evaluating CE initiatives by systematically describing key dimensions of engagement and impact [11,12]. Originally developed for evaluating public health and clinical interventions, RE-AIM has been increasingly recognized as a useful tool for community-based research, as it allows for the examination of both individual- and organizational-level factors influencing program success [12]. Partnering visualization tools with the RE-AIM framework could aid in the communication of a multitude of complex CE program outputs and outcomes, which can make it a promising application for evaluating CE activities with CTSAs [13]. Therefore, RE-AIM provides the structure and level of flexibility that assesses individual, participant level, and organizational programmatic factors, making it a good fit for CE initiatives [12].

Evaluating CE programs often involves working with multiple data types collected across varying contexts, making it challenging to quickly monitor and interpret programmatic effects. Visual tools can play a role in addressing these difficulties by helping evaluators compare outputs/outcomes across domains, populations, and settings. Radar charts, what we refer to as “Net Effects Diagrams” in this paper, offer a way to display multivariate data in a way that is both accessible and actionable [14]. When paired with standardized scoring methods, these visualizations can provide both summative insights (i.e., ‘Does it work?,’ “Is it effective?”) and formative feedback that supports program planning, monitoring, and improvement.

This paper outlines a generalizable method for operationalizing programmatic evaluation to produce and monitor outcomes, using our community-based educational workshops as an illustrative case in the results section. This structured, step-by-step approach, centered around the RE-AIM framework and supported by a standardized scoring system, is designed to be transparent and replicable across diverse program contexts and settings. This method is a structural proof-of-concept that can be readily adapted for unique contexts (e.g., setting, program, organizational structure, program purpose, etc.) outside of the example provided in this paper.

## Methods

**Figure 1.**
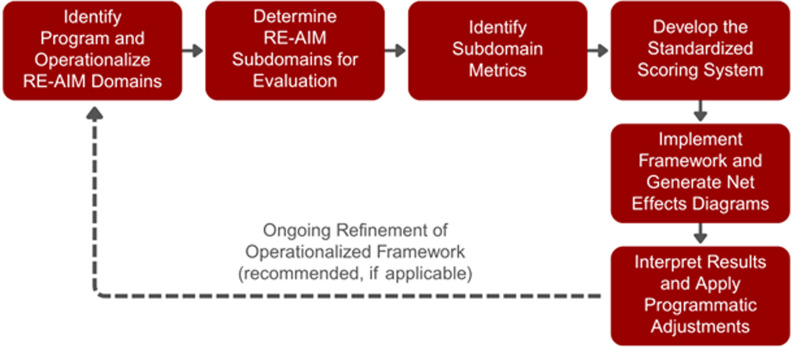
Seven-step process for operationalizing RE-AIM. RE-AIM, reach, effectiveness, adoption, implementation, and maintenance.

This methods section outlines a seven-step, structured process for operationalizing RE-AIM, through standardized scoring and Net Effects Diagrams, to support customizable, scalable, and replicable programmatic and context specific evaluation (Figure 1).

### Step 1: Identify program and operationalize RE-AIM domains

The first step involves selecting the program to be evaluated and defining each RE-AIM domain in operational terms. Programs should be selected based on their strategic relevance, implementation maturity, and availability of evaluation data. Each domain should be interpreted in a way that aligns with the program’s goals, intended impact, and contextual factors (e.g., setting, population served, or implementation approach). For example, *Reach* may refer to the number of individuals engaged, proportion of the target population engaged, and/or degree of demographic alignment between participants and intended audiences. These definitions should be developed collaboratively with program staff and key stakeholders to ensure alignment with both implementation goals and community priorities.

### Step 2: Determine RE-AIM subdomains for evaluation

Domains can be further divided into relevant subdomains to increase specificity and enhance the actionability of findings. Subdomains allow evaluators to focus on meaningful components of each domain that better reflect implementation realities and measurement priorities. Some examples include:

- Reach: frequency of programming, participant attendance, representativeness of attendees
- Effectiveness: increases in participant knowledge, satisfaction, and self-efficacy
- Implementation: fidelity to evidence-based practices, perceived responsiveness to community needs

The number of subdomains may vary depending on the domain and the program. Some may only require one subdomain, while others may benefit from multiple. Subdomains are not always necessary in every domain. For instance, *Maintenance* may be evaluated through sustained outcomes over time or program continuity across years and may not require further breakdown. Whereas the domain *Adoption* can be conceptualized as differences in the *Reach*, *Effectiveness*, and *Implementation* outputs between contexts, such as site type, implementation strategy, or time frames.

### Step 3: Identify subdomain metrics

For each subdomain, evaluators must identify one or more metrics to capture performance. These metrics should be relevant to program goals, interpretable by implementers and stakeholders, and consistently collected across time and groups. Metrics can include a variety of quantitative indicators, such as Likert-scale responses (i.e., satisfaction, confidence), pre/post assessments (i.e., knowledge gain), observational or staff-reported ratings (i.e., outreach efforts, participant engagement), and attendance or frequency counts.

When a single metric does not fully represent a subdomain, multiple indicators can be combined to reflect its broader scope. Metric identification should be grounded in the program’s logic model or objectives, and can draw from existing tools, adapted instruments, or newly developed items that align with the evaluation and program’s needs. Metrics can comprise various perspectives (e.g., participant and facilitator feedback) as a form of triangulation to increase credibility and validity in the findings by cross-verifying information.

### Step 4: Develop the standardized scoring system

The choice of central tendency should reflect data distribution as means provide more granular differentiation but are sensitive to outliers, whereas medians resist skewness but may obscure meaningful variation. Outliers should also be assessed, as they can either represent meaningful real-world differences or distort values/outputs, and evaluators should document whether they are included, excluded, or analyzed separately. Reporting measures of spread such as standard deviation or interquartile range alongside any average scores used is recommended to provide additional context and avoid masking variability.

To support comparability and visualization across subdomains and domains, all selected metrics should be converted to a standardized scale. A common 1-to-5 scoring scale is recommended, where 1 indicates the lowest performance and 5 the highest. Standardization enables aggregation and supports intuitive visual interpretation. The scoring process generally involves:

1. Reviewing historical data or relevant benchmarks for each metric.
2. Drafting preliminary score thresholds (e.g., using quintiles or meaningful performance benchmarks).
3. Facilitating a review with subject matter experts or experienced program staff to validate cutoffs.
4. Adjusting thresholds based on contextual insights, ensuring the scores reflect real-world differences in performance.
5. Documenting the finalized scoring system along with the rationale for each range.

Once the general process is established, it can be applied to individual metrics to translate raw data into standardized scores. This step is crucial for ensuring that results from diverse measures can be interpreted on the same scale. It is important to note that scoring thresholds are not universal and should be tailored to the context of the program and refined over time as goals evolve or more data becomes available, for example as when measuring percent change (0.0–9.9% change = score of 1; 10.0–19.9% = 2; 20.0–29.9% = 3, etc). When applying this approach, evaluators should carefully consider how to summarize and compare data between groups.

### Step 5: Implement framework and gnerate net effects diagrams

Standardized scores can now be calculated for each metric. For subdomains with multiple metrics, the individual standardized scores are averaged to generate a subdomain average score. Scores were visualized with Net Effects Diagrams to aid interpretation and enable comparisons across *Adoption* level variants (e.g., site type, implementation strategy, or time frame). These diagrams plot subdomain scores along axes extending from a central point. Each comparative program variant (e.g., between phases of a pilot program), group (between sites), or time frame (quarter vs. quarter) can be displayed on the chart, making differences easy to identify and interpret. These diagrams can be created using common tools like Microsoft Excel and are helpful in communicating patterns across programs thus informing programmatic assessment and decisions.

### Step 6: Interpret results and apply programmatic adjustments

Teams should engage in regular review sessions to interpret Net Effects Diagrams and subdomain scores. Comparing results across RE-AIM domains can help uncover relationships (e.g., a high-reach program with low effectiveness) and inform adjustments. If the program underperforms in any domain, examining subdomain results can clarify shortcomings and highlight variability that suggests inconsistent quality.

Discussions with program staff and stakeholders can help generate ideas of how to improve. Responsive adaptations could include modifying outreach strategies if *Reach* is low, updating content or delivery methods if satisfaction or knowledge scores are poor, or enhancing facilitator training if *Implementation* fidelity scores drop. Visualizing the outputs/outcomes supports transparency, shared understanding, and use of evaluation data in decision-making, which aids in communicating findings to a range of audiences.

### Step 7: Ongoing refinement of operationalized framework

Evaluation frameworks should be viewed as dynamic tools that evolve alongside the programs they assess. As program goals shift, implementation strategies change, new staff is hired, or new audiences are engaged, the operationalized RE-AIM framework should be revisited to ensure continued alignment and relevance. Given the active participation of program staff in the development of this approach and the assessment of programmatic performance, efforts to “calibrate” staff observations and ensure a reasonable level of inter-rater reliability are required.

This step involves periodically reviewing and refining each element of the approach, including operational RE-AIM domain definitions, subdomains including how they are defined, selected metrics, and scoring thresholds. Programs that operate long term, scale to new settings, or undergo significant changes in content or implementation would particularly benefit from regular reassessment and ongoing calibration (e.g., annually or following major programmatic shifts). These reviews help ensure the framework remains responsive to changing conditions and continues to reflect both implementation goals and community values, while also allowing for benchmark comparisons over time. Any updates to the framework should be clearly documented and include the rationale for the change and its implications for longitudinal comparisons.

Stakeholder input, including that of program staff, participants, and community members, should inform these refinements. Qualitative insights or emergent themes from community feedback can highlight blind spots or unintended consequences in the evaluation approach that may not be captured through quantitative metrics alone. Incorporating ongoing refinement as a component of the approach enhances adaptability, ensures sustained alignment with program objectives, and supports long-term effectiveness of the evaluation system.

## Results

This section presents the results of applying the seven-step approach to operationalize the RE-AIM evaluation framework by using our community-based health education workshops as an illustrative example. Workshops were delivered in both English and Spanish, providing an opportunity to assess program performance across language groups utilizing the standardized scoring system and generating Net Effects Diagrams. The survey data presented in this section were collected between January and September 2024 from a total of 81 workshops. There were 26 workshops delivered in English to a total of 215 participants, 134 of whom completed the pre and post surveys, and 55 workshops delivered in Spanish to a total of 1,103 participants, 725 of whom completed the pre and post surveys. It should be noted that not all who completed the surveys answered every question either intentionally or unintentionally, therefore there is variation in item-level response rates.

### Step 1: Identify program and operationalize RE-AIM domain

The workshops were designed to improve participants’ knowledge and confidence on health topics related to nutrition, chronic conditions, and mental health, among others. Workshops were tailored to and delivered across multiple sites. RE-AIM domains were defined for related CE activities in collaboration with program staff and leadership, and are as follows:

- Reach: The extent to which the intended population is engaged in programming and services*.
- Effectiveness: The extent to which programming and services* achieve the intended short-, medium-, and long-term goals as stated in the logic model.
- Adoption: The transferability and scalability of programming and services* to other settings (e.g., expand partnerships) and participants (e.g., providing activities in English and Spanish). The innovation and uptake of service models (e.g., place-based approaches) and innovations (e.g., dissemination efforts).
- Implementation: The extent to which evidence-based practices and community perspectives are used to shape programming and services* while testing innovative service and dissemination models.
- Maintenance: The extent to which programs, services, and service models are sustainable over time (e.g., partnerships, examples of institutionalization, additional funding sources, integration into existing institutions/programs/ leadership/staffing, and/or changes in institutional policies/practices).

* SC CTSI CE programming and services include, but are not limited to, educational offerings, connections to care, and clinical trial promotion and participation.

### Step 2: Determine RE-AIM subdomains for evaluation

Subdomains were then identified in collaboration with program staff and members from our leadership and executive teams to reflect the program’s key implementation components, outcomes of interest, and available data sources (Table 1). These subdomains were meant to operationalize each RE-AIM domain in a way that would reflect workshop-specific design and implementation features.

**Table 1.** SC CTSI CE Operationalization of RE-AIM Subdomains

Domain: subdomain	Metric	Description
**Reach:** Frequency	Average monthly number of workshops offered	The average number of workshops offered per month.
**Reach:** Attendance	Perceived outreach effort by CE staff	CE staff perception rating of the relative effort made to do outreach for and promote the workshop.
	Average attendance per workshop	The average number of participants per workshop.
	Perceived participant turnout by CE staff	CE staff perception rating of the level of participant turnout to the workshop based on outreach and recruitment efforts.
	Characteristics of participants	CE staff perception rating of the degree to which the characteristics of the participant group of a workshop aligned with the expected characteristics based on the recruited for the workshop.
**Effectiveness:** Immediate Reaction	Participant satisfaction	The average participant rating on post-workshop participant satisfaction statements on a 4-point scale.
	Perceived level of participant engagement by CE staff	CE staff perception rating of the level of engagement by participants.
**Effectiveness:** Short-Term Goals	Participant change in knowledge	The average percent change in participant knowledge scores between pre-workshop and post-workshop.
	Participant perceived knowledge improvement	The average participant rating of their perceived knowledge improvement.
	Participant self-efficacy/ confidence levels	The average participant rating of their confidence levels in using what they learned and/or performing a skill learned.
	Participant intention to apply workshop content in their own lives	The average participant rating for their intention to incorporate what they learned from the workshop into their lives.
**Effectiveness:** Medium-Term Goals	Participant application of knowledge/skill	The average participant rating of how they have applied what they learned from a workshop.
	Participant impact	The average participant rating in how much the participation in a workshop has improved their health/lives.
**Implementation:** Evidence-Based	Level of evidence-based content rating	CE staff perception rating of the level of evidence-based information/ practices incorporated in the workshop.
**Implementation:** Responsiveness to Community Needs	Expert reflection/ staff perceptions - how did it go?	CE staff perception rating of how they felt about the workshop overall.
	Level of cultural relevancy	CE staff perception rating of how culturally relevant the workshop content and discussions were for the intended audience.

SC CTSI = Southern California Clinical and Translational Science Institute; CE = Community Engagement; RE-AIM = Reach, Effectiveness, Adoption, Implementation, and Maintenance.

### Step 3: Identify subdomain metrics

Metrics were identified for each subdomain based on existing evaluation tools. Items were then added to existing data collections tools as needed to ensure each subdomain was supported by one or more relevant metrics. Pre-workshop surveys were administered to participants to collect demographic information and test their knowledge on the workshop topic (i.e., pre-workshop knowledge score). Post-workshop surveys were administered to participants to reassess their knowledge (i.e., post-workshop knowledge), along with questions to assess their self-efficacy/confidence levels, satisfaction, and intention to apply what they learned. Attendance sheets were used to log attendance. CE staff facilitating the workshops completed a post-workshop facilitator feedback form where they rated statements related to their perceived understanding of outreach efforts, participant engagement, and effectiveness of the workshop. All data were collected and managed using REDCap electronic data capture tools hosted at Yale University [15,16]. An outline of the specific metrics for each subdomain can be found in Tables 2 and 3.

**Table 2.**
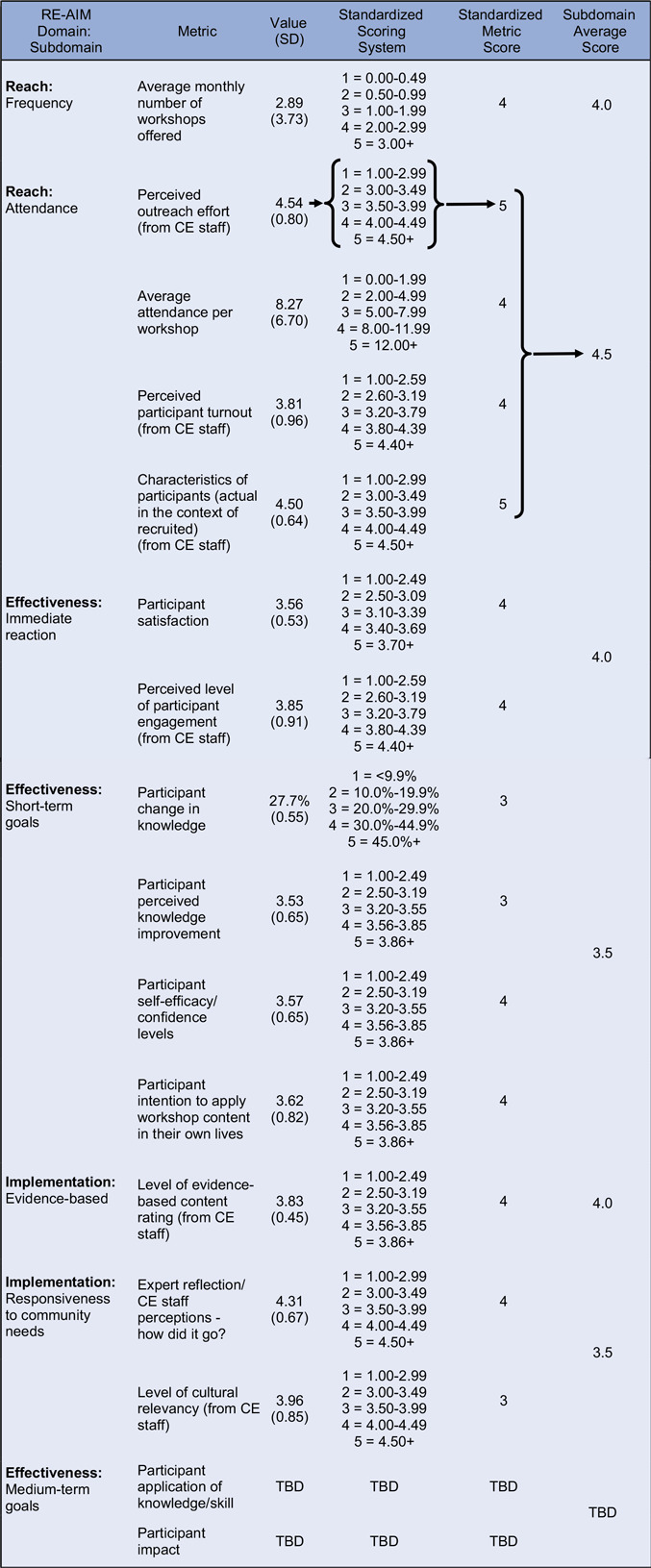
Operationalized outputs for English-language workshops (January - September 2024)

RE-AIM = Reach, Effectiveness, Adoption, Implementation, and Maintenance; SD = standard deviation; CE = Community Engagement; TBD = To Be Determined.

**Table 3.**
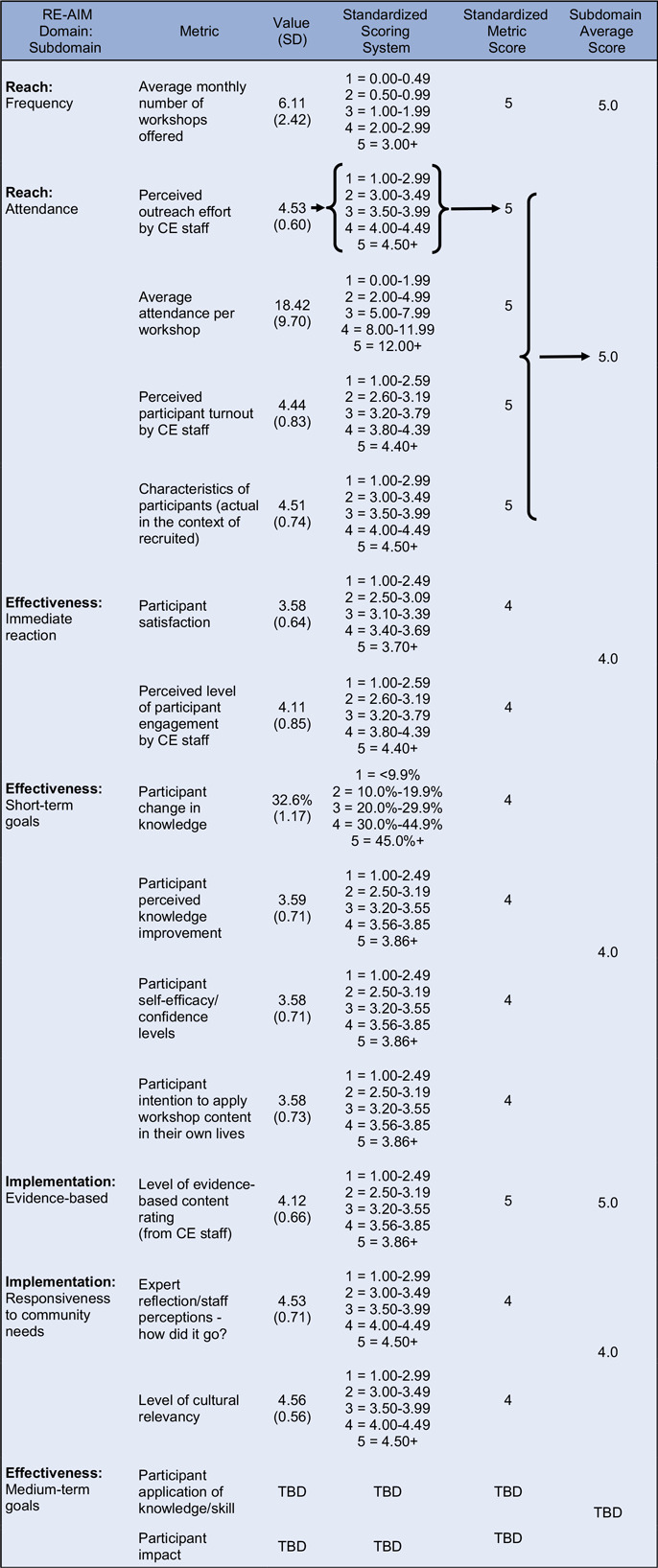
Operationalized outputs for Spanish-language workshops (January - September 2024)

RE-AIM = Reach, Effectiveness, Adoption, Implementation, and Maintenance; SD = standard deviation; CE = Community Engagement; TBD = To Be Determined.

### Step 4: Develop the standardized scoring system

We examined the distributions of data to determine appropriate measures of central tendency for comparing English- and Spanish-language groups. Given the small, variable sample sizes, we used mean values as the primary summary statistic, as they offered clearer group differences than medians, which in some cases (e.g., knowledge gain percent change) were uniformly 0.00% and thus uninformative. We did not exclude outliers from the central tendency analysis, as they represented meaningful participant experiences that we wanted to capture. Standard deviations are reported alongside mean values in Tables 2 and 3 to provide context about variability.

We then developed a standardized scoring system for each subdomain metric, allowing for consistent interpretation, comparison, and aggregation of results across workshops and between participant groups. Standardized scoring thresholds for each metric were developed iteratively, incorporating historical workshop data to identify performance distributions and input from senior staff. Using historical data, we generated preliminary thresholds based on percentile groupings or natural breakpoints in the data, while also being mindful of ceiling effect threats. For newly developed metrics, we used a combination of informed estimation and practical experience to determine initial scoring thresholds.

The historical data distributions, drafted thresholds, and potential case examples were then reviewed by senior staff. During this review process, they were asked to evaluate the thresholds and provide feedback based on the following criteria.

- Real-world interpretability: Do the thresholds meaningfully distinguish levels of program performance from an implementation perspective?
- Actionability: Would a score in a particular range prompt a different programmatic response or improvement strategy?
- Alignment with expectations: Do the thresholds reflect what staff would consider low, average, or high performance based on their field experience and program goals?

Based on this feedback, we adjusted thresholds to better reflect implementation realities and added narrative justifications for each score range to improve transparency and internal replicability. For example, for the participant change in knowledge metric, the threshold for a score of 3 was raised from 10 to 20% change after staff indicated that a 10% increase was commonly achieved and thus not representative of “average” metric performance. Similarly, satisfaction scores were reclassified so that a score of 5 reflected not just high averages but consistently high individual ratings across all satisfaction items. Tables 2 and 3 outline the standard scoring ranges for each metric. This combined quantitative-qualitative process ensured that the scoring system was both evidence-informed and grounded in practical experience and application, increasing its utility for program improvement and ease of internal replication.

### Step 5: Implement framework and generate net effects diagrams

Workshop data was organized, separated by language, and analyzed using Microsoft Excel. Participant- and staff-reported data were compiled across all selected metrics and organized by RE-AIM subdomains (Tables 2 and 3). Raw metric values (e.g., percent change in knowledge, Likert-scale ratings) were converted into standardized scores based on the validated scoring thresholds described in Step 4.

For subdomains composed of multiple metrics, we first calculated individual metric based standardized scores on a 1-to-5 scale. We then averaged them to create an overall subdomain score, allowing us to summarize performance across multiple indicators within each RE-AIM domain. This approach enabled consistent aggregation across both ELW and Spanish-language workshops (SLW) while maintaining sensitivity to nuances of each contributing metric.

**Figure 2.**
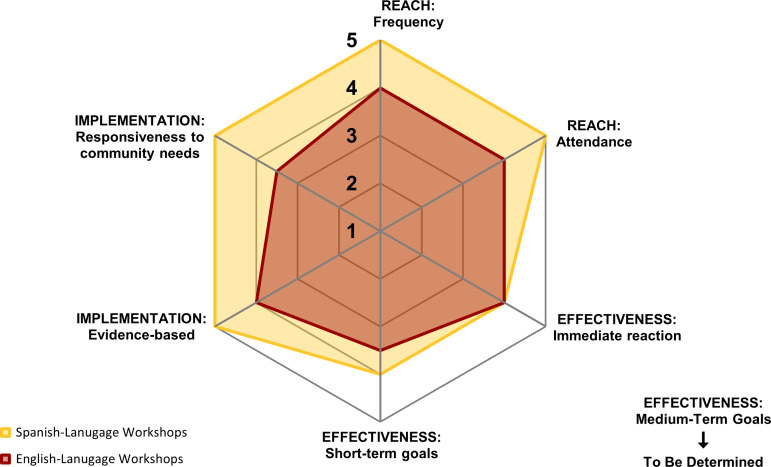
Net effects diagram comparing operationalized outputs for english-language workshops versus spanish-language workshops.

All data processing was conducted using basic formulas and logical functions, which allowed for transparent calculations, ease of error checking, and replicability. We generated Net Effects Diagrams within Excel to visually compare the performance of ELW versus SLW across RE-AIM subdomains (Figure 2). By plotting subdomain scores for each group along the same axes, we could visualize how program groups differed from one another (e.g., higher “Reach: Attendance” but lower “Effectiveness: Short-term goals”) and begin interpreting patterns to guide future programming.

### Step 6: Interpret results and apply programmatic adjustments

We interpreted the Net Effects Diagram results to identify key patterns in program performance and used those insights to inform potential programmatic refinements. The Net Effects Diagrams that we generated provided a clear visual representation of differences in program implementation and outcomes between ELW and SLW, supporting both internal team reflection and broader stakeholder discussion.

Key Patterns and Insights:

- Reach: SLW had slightly higher subdomain scores for frequency of workshop delivery and participant attendance. This suggests greater consistency and participation compared to ELW.
- Effectiveness: SLW had higher scores in short-term effectiveness, including knowledge gain, perceived knowledge improvement, confidence in applying information, and intention to use the material, indicating a stronger immediate impact on participants in the SLW.
- Implementation: Scores for facilitator responsiveness to participant needs and adherence to evidence-based practices from SLW were higher, suggesting stronger alignment between delivery methods and participant expectations.

The combined results displayed in the Net Effects Diagram highlighted a pattern of stronger performance for SLW, particularly in the *Reach* and *Implementation* domains. These differences were discussed in depth during a CE team review session, where we used the visual outputs to identify areas for adaptation in the implementation of ELW. Another adjustment identified was to better tailor the content for ELW, as we recognized the opportunity to further adapt materials to reflect the specific needs, experiences, and contexts of the participants. This might include revising examples, emphasizing relevant topics, and incorporating more community-specific language and examples.

Given that SLW were offered in more established community partner settings with long-standing relationships and ELW were part of a recent expansion into new communities, these findings aligned with team expectations. The use of the operationalized RE-AIM framework helped these nuances emerge and provided a clear path forward for continuous quality improvement and targeted program refinement. As these data-driven programmatic adjustments are implemented, the measurement process can be replicated, creating multiple Net Effects Diagram “snapshots” that may aid in understanding what drives changes in overall effectiveness over time.

### Step 7: Ongoing refinement of operationalized framework

Our team’s next step will be to conduct a structured reflection process to assess the clarity, utility, and continued relevance of the current operationalized RE-AIM framework as applied to our health education workshops. This will include a focused review of how we defined each RE-AIM domain, the usefulness and interpretability of our selected subdomains, and the appropriateness of scoring thresholds used to standardize and compare outcomes. Our team will consider whether the current framework sufficiently captures what matters most to both community participants and partners. Key questions for discussion will include:

- Do the current domain and subdomain definitions remain aligned with how the workshops are evolving?
- Are any important aspects of workshop quality, accessibility, or impact missing from the current framework?
- Do the scoring thresholds accurately reflect meaningful differences in performance?

While the framework supported useful comparisons and decision-making, there may be areas where further refinement could improve clarity and alignment. For example, some subdomain metrics may require more precise definitions, and certain scoring thresholds may need to be recalibrated as more data become available. The future iterations of the framework may involve adjusting metric definitions or adding new subdomains based on emerging implementation priorities, revising score ranges to better reflect updated benchmarks or community expectations, and incorporating additional qualitative feedback loops from community participants and facilitators. Any changes made will include the rationale and implications for comparison over time, supporting transparency and continued usability. This refinement process will reflect our team’s commitment to maintaining a responsive and evidence-informed evaluation approach that evolves alongside the program and the communities it serves.

## Discussion

The paper presents and demonstrates a structured, replicable step-by-step evaluation approach grounded in the RE-AIM framework that supports rigorous, stakeholder-informed evaluation. Although originally designed for use in community-engaged research and implementation settings, it has the potential to be applied in other fields and settings. By integrating domain specific subdomains, scoring standardization, and visualization through Net Effects Diagrams, this approach allows for consistent assessment and actionable comparisons across diverse programs or delivery contexts. It also allows for data-driven assessments of what is working, where, and why, demystifying the challenge of answering, “how do we know?.” The application of this method to our health education workshops demonstrated its feasibility and highlighted how the structured use of RE-AIM can reveal nuanced performance patterns and guide targeted program improvements and overall assessment of programming.

### Structuring RE-AIM and creating comparable metrics

RE-AIM’s broad domains can be difficult to apply in settings where data is: (1) sourced from multiple tools and levels; (2) varied in type; (3) short-term in nature; and/or (4) embedded in dynamic community contexts. Our approach addresses this by defining tailored subdomains, grounded in local implementation realities and refined through engagement with the team. This enables consistent monitoring while preserving the contextual nuances critical to CE work. In our workshops, this process helped translate abstract constructs like “Effectiveness” into specific measures, such as participant-reported confidence levels and increases in knowledge. Our subdomains revealed key differences between ELW and SLW that would have been obscured by high-level metrics alone.

The development of standardized subdomain scores allowed for the comparison of various data types and from participants and facilitators across workshop topics, locations, and populations, an essential step for CE teams managing multiple programs. This scoring system offers a structured rubric system and a more meaningful understanding of the key differences between groups that accommodates program and implementation specific realities. For example, SLW consistently outperformed ELW in measures of “Reach: Attendance” and “Effectiveness: Short-term goals.” Rather than interpreting this as a failure of ELW, our scoring system contextualized the results, prompting useful questions about outreach strategies and content relevance. For example, our team noted that many of the participants in SLW were reached through trusted community and faith-based partners. To improve *Reach* for ELW, the team recommended expanding recruitment efforts across our communities to include similar trusted venues. In this way, scoring supported both interpretation and action.

### Visual tools for shared understanding and decision-making

The use of Net Effects Diagrams to visualize RE-AIM scores across subdomains further enabled accessible and data-informed decision-making. These tools helped our team quickly identify where programs were excelling, where adjustments or help may be needed to facilitate collaborative interpretation across roles and expertise levels. When we applied the diagram to compare groups, the visual differences helped generate immediate, understandable insights, such as the greater engagement observed in SLW and the need to revisit outreach strategies for ELW. This reinforces the value of clear, interpretable tools for facilitating feedback.

### Limitations and considerations

Several limitations and considerations should be noted about this approach and opportunities for adoption. First, CE work often relies on self-reported outcomes that can be limited in capturing long-term behavior change and can introduce the potential for response bias. However, self-reported data is still an acceptable and commonly used data collection method to evaluate activities like health education workshops that have a focus on engagement, knowledge comprehension, and short-term outcomes. Future iterations of our CE evaluation will incorporate follow-up assessments to further understand medium-term outcomes.

Second, small sample sizes, particularly in subgroup comparisons, can limit generalizability. However, the approach’s value lies in its ability to identify trends early and guide strategy adjustments, rather than to establish causal effects. Third, while the use of mean values as our measure of central tendency and the application of them for creating standardized scores allowed for more granular between-group comparison, this process can obscure variability within the data and may be sensitive to outliers. Future applications of this approach will consider alternative strategies such as median-based scoring, trimming outliers, or sensitivity analyses to improve robustness of comparisons. Others applying this approach should carefully review the distribution of their data and select central tendency measures that best fit their context and analytical objectives.

While this approach provides a structured process for identifying and defining domains, subdomains, and metrics, the development of rating scales and parameters remains context-specific and may vary across applications. While this flexibility allows for tailoring to program priorities and data availability, the approach is not intended to generate universally standardized measures and is best understood as a structured guide rather than a fixed standard. Future applications across multiple programs will refine common parameters to enhance comparability.

Importantly, this approach is not specific to workshops and can be applied to other CE activities where systematic monitoring and adaptation are needed (e.g., community-academic partnerships, resource navigation, or capacity building activities). Its blend of structure and flexibility makes it especially well-suited to the varied and evolving landscape of CE and other fields [17]. We also recognize that the reporting of this proof-of-concept represents its use for one type of activity administered by our team who also developed this operationalized approach. This approach has begun to be implemented elsewhere within our institute to help develop the formal and developmental evaluation of our institute’s research trial recruitment dashboard. It has helped them, from a programmatic approach, to understand what metrics needed to be assessed to evaluate effectiveness over time.

### Implications and future directions

This approach offers a guide for CE programs seeking to evaluate and improve their efforts systematically, rigorously, and comprehensively. Our application of Net Effects Diagrams shows how the operationalized RE-AIM framework can be practically implemented, interpreted, and adapted to inform meaningful improvements to CE program design and delivery. This ultimately supports more impactful and responsive engagement with the communities we serve.

Future directions include scaling this approach across our other CE activities, expanding outcome tracking to include longer-term measures, and integrating results into more dynamic database systems and interactive dashboards to enhance accessibility and responsiveness. As this approach expands beyond the proof-of-concept phase, we will continue to work collaboratively and incorporate more direct input from our stakeholders. To support evaluation capacity building across the CTSA network and beyond, this approach could be disseminated as a toolkit or adapted into training modules and interactive webinars. Such resources would enable broader uptake and customization of the method across settings while reinforcing shared learning and cross-site evaluation infrastructure.
